# Schools as a Source of Mental Health Literacy: Adjusting and Validating a Mental Health Literacy Scale

**DOI:** 10.1177/15248399231161090

**Published:** 2023-03-20

**Authors:** Hanne Nissen Bjørnsen, Gunnar Bjørnebekk, Christian Brandmo

**Affiliations:** 1 Norwegian University of Science and Technology, Trondheim, Norway; 2 University of Oslo, Oslo, Norway

**Keywords:** mental health literacy, adolescent, mental health, positive mental health, mental health measurement, instrument validation, mental health learning, mental health in school, mental health promotion

## Abstract

Over the last decade, there has been a growing interest in mental health literacy (MHL) in health promotion, largely motivated by increased awareness of MHL as a modifiable determinant of mental health. Accordingly, MHL has been associated with the health-promoting school approach emerging over the last 20 years. To succeed in promoting MHL, it is of vast importance to evaluate working strategies and interventions to address MHL using validated instruments. The current study describes the revision and psychometric testing of a modified version of the 10-item adolescents’ positive MHL measure, the MHPK-10, the only identified instrument measuring adolescents’ positive MHL. The MHPK-10 was adjusted to address the previously documented ceiling effects and was further optimized for use in schools by reworking it to measure learning rather than self-reported knowledge, becoming the new nine-item Mental Health Learning Scale (MHLS-9). The MHLS-9 was tested on a national sample of *N* = 2,012 Norwegian ninth graders. Data were analyzed by confirmatory factor analysis (CFA) and tests of reliability and validity. The revised CFA model for the MHLS-9 showed an improved fit over the original CFA model for the MHPK-10. The MHLS-9s’ CFA model revealed excellent factor determinacy (.95) and scale reliability (ω = .91). Thus, the MHLS-9 is an improved measure for the positive component of MHL for use in school settings, enabling researchers and practitioners to evaluate and focus positive MHL interventions in schools using a short, reliable, and valid measure for adolescents’ learning about the factors promoting good mental health.

## Background

Adolescent mental health has over time received considerable attention as an important issue to be addressed in schools, health care, public health, and society as a whole, taking on a central focus the last several years due to the COVID-19 pandemic (Fazel & Hoagwood, 2021). Mental health literacy (MHL) has increasingly been acknowledged as a central concept in effectively addressing and promoting adolescent mental health (Kutcher et al., 2016). MHL is a multifaceted construct originating in health literacy (HL), broadly referring to the knowledge and abilities necessary to benefit mental health (Jorm, 2012; Kutcher et al., 2016; Wei et al., 2017). Over the last decade, there has been a growing interest in MHL in health promotion among adolescents, largely motivated by the increased awareness of MHL as a modifiable determinant of health, an outcome of health education with the potential to benefit adolescents’ current and future mental health (Kutcher et al., 2016). MHL has been defined as comprising four distinct and related components: understanding how to obtain and maintain good mental health (positive MHL), understanding mental disorders and their treatments, decreasing stigma, and enhancing help-seeking efficacy (Kutcher et al., 2016, p. 567), where Component 1, positive MHL or how to obtain and maintain good mental health, was recently recognized as a determinant of a population’s mental health and considered a resource for mental health throughout the lifespan (Carvalho et al., 2022).

Health and education are strongly related (Langford et al., 2014), where HL, including MHL, has been associated with the health-promoting school approach that has been emerging over the last 20 years (Paakkari & Okan, 2019). Furthermore, the need to look beyond the health sector to promote mental health is gaining recognition (Butler et al., 2022), where schools are widely being identified as an appropriate and vital setting for promoting adolescent mental health (O’Connor et al., 2018). In addition, most adolescents attend and spend a considerable amount of their time at school. As such, schools play a key role in adolescents’ development, from, for example, mental health and peer relationships to academic attainment, and they serve as an ideal location for promoting and learning about obtaining and maintaining good mental health (Butler et al., 2022). Nevertheless, schools and health care traditionally exist as separate systems, despite overlapping responsibilities and interests, one of which is adolescent mental health (Colizzi et al., 2020; O'Reilly et al., 2018). In Norway, public health and life skills were recently introduced as integral parts of the school curriculum in the 2020 curriculum renewal (Utdanningsdirektoratet, 2020). One central objective of the curriculum renewal was to teach competence that promotes sound physical and mental health and to provide students with the knowledge and tools to make responsible life choices, that is, HL and MHL, where positive MHL relates particularly well to the curriculum renewal objectives.

To succeed in promoting positive MHL, both in schools and in health care settings, it is of vast importance to evaluate working strategies and interventions addressing MHL using validated instruments. However, MHL measurement, and particularly the positive aspects of MHL linked to the promotion of good mental health, is an under-researched area among adolescents. To the authors’ knowledge, only one measure of positive MHL among adolescents is available: the 10-item Mental Health-Promoting Knowledge (MHPK-10; Bjørnsen et al., 2017).

The MHPK-10 is a self-administered instrument developed in 2017 to measure adolescents’ positive MHL in the context of school health services (Bjørnsen et al., 2017). The instrument is customized to school health services work, and it has a ceiling effect (Bjørnsen et al., 2017).^1^ However, only minor modifications are thought needed to address the ceiling effect and to adapt the MHPK-10 to identify and evaluate the mental health-promoting work grounded in the school curriculum renewal.

## Aims

This study aimed to address the previous ceiling effect and adapt the MHPK-10 to measure the MHL within the schools’ curriculum. More specifically, the aim was to adjust the MHPK-10 to measure adolescents’ perceived *learning of MHL at school* rather than the subjective knowledge levels of factors promoting good mental health, as well as to investigate the psychometric properties and validity of the modified instrument among a wide selection of Norwegian ninth graders and to test the modified instrument’s factor structure, reliability, and validity.

## Method

### Participants and Procedure

This study is based on data from a national evaluation of Norway’s 2020 curriculum renewal. The participants were *N* = 2,012 ninth graders (50.1% male) from 61 public schools across the country. The study used a complex sampling design, meaning schools were selected, but all students in the population had an equal probability of being drawn. The sampling procedure also included an adjustment for geographical location, so the country’s various regions were proportionally represented. In addition, if a school declined to participate, the next school on the population list in the region was selected. At the selected schools, 48% of the chosen students participated. There are two main reasons for dropout, and both are related to the COVID-19 pandemic: first, the schools were under heavy pressure to complete the required academic activities due to several shutdowns and extensive digital teaching, and participation in a survey was given low priority. The second reason was that we struggled to get parents to respond to the consent form, despite significant information, work, and several reminders. Due to dropout for various reasons, we hesitate to claim that the survey participants are representative of the national population of ninth graders. We nevertheless believe the variation in student characteristics is satisfactory for validating a measurement instrument.

The data collection for the main study included measurement of 30 different concepts in total, seven of which were included in the current study. The data collection took place digitally by the students logging into an online survey while at school. Teachers administered the data collection according to standardized procedures (e.g., instructions and which questions could be answered).

### Ethics

The current study is approved by the Norwegian Centre for Research Data (NSD) (Registration number NSD 766872/2020). The students and parents provided written informed consent prior to the data collection, and the study was conducted in compliance with the Declaration of Helsinki. Data were stored with the Services for Sensitive Data (TSD) at the University of Oslo in compliance with the General Data Protection Regulation.

### Study Variables

All measures used in this study were adapted for use among adolescents from previously validated scales and piloted in a smaller sample (*n* = 230) prior to the main study (*N* = 2,012), where data for the current study were collected.

#### Mental Health Learning Scale Based on the MHPK-10

The MHPK-10 is a self-administered instrument developed in Norway to measure adolescents’ positive MHL in the setting of school health services. The scale consists of 10 items assessed on a 6-point scale from 1 = *completely wrong* to 5 = *completely correct*, including the option *I don’t know*. The scale covers three core theoretical components of good mental health originating in the basic psychological needs theory: relatedness, knowledge, and competence (Bjørnsen et al., 2017). A recent systematic review performing a quality assessment of HL instruments rated the quality of the MHPK-10 as excellent (Tavousi et al., 2022).

The current study introduces the new nine-item Mental Health Learning Scale (MHLS-9), which is an adjustment of the MHPK-10 that aims to address the ceiling effects and to optimize the measure further by reworking the introduction sentence of the instrument to measure *learning at school* rather than self-reported knowledge. Furthermore, one item (Item 10) was removed, as it asked about experiencing school mastery and was considered redundant in the new contextualization. The remaining nine items were retained as the original scale. The introduction sentences were changed from the original: “Here are 10 statements about things that can be important for good mental health. On a scale from 1 to 5, how correct is each statement?” to “To what extent have you learned skills at school that have made you better at the following” <e.g., believing in yourself>. The nine items were answered on a 5-point Likert-type scale ranging from 1 = *to a very small extent* to 5 = *to a very large extent*, and the option *I don’t know* was removed from the original scale.

#### Well-Being–Positive and Negative Affect

Positive and negative emotions were assessed with eight items from the Norwegian Counties Public Health Survey (Nes et al., 2018). The response format and selection of items conform to Organization for Economic Cooperation and Development (OECD) guidelines in the subjective well-being literature, and the scale’s reliability and validity have been documented in normal populations in large-scale Norwegian studies (Nes et al., 2022). The scale had three items expressing positive emotions (calm and relaxed, happy, and engaged) and five items expressing negative emotions (worried, down or sad, irritated, lonely, and anxious). The students were asked the following: “Think about how you have felt over the last 7 days. To what extent were you < e.g., sad>?” The questions were answered on a 10-point anchored scale ranging from 1 = *not at all* to 10 = *all the time*.

#### Sense of Personal Control

The Norwegian five-item perceived constraint facet of Pearlin and Schooler’s Mastery Scale (1978) was used to assess sense of personal control (e.g., “When I face problems in my life, I often feel helpless”), with each item rated on a 10-point anchored scale ranging from 1 = *completely disagree* to 10 = *completely agree*. For this scale, the item scores were reversed in the analyses, with high scores indicating a greater sense of personal control. Clench-Aas and colleagues (2017) validated the five-question version in a large representative Norwegian sample.

#### Productive Coping Strategies

To assess productive strategies for coping with challenging situations in an academic context, we used six items from the Adolescent Coping Scale (ACS; Frydenberg, 2018). The original version includes 90 questions and assesses 18 different coping strategies. Factor analysis was used to group the strategies into objectives for coping styles (Frydenberg & Lewis, 2009). Exploratory factor analysis on the 18 strategies normally results in three factors: task-focused coping style, where strategic approaches are used to tackle tasks; getting help from others; and nonproductive coping style. In this study, three items assessed task-focused strategies (e.g., “Initiate extra training/training to master similar situations”) and three items assessed help-seeking strategies (e.g., “Contact someone who can help me deal with such situations [teacher, parent, counselor, or classmate]). The students received the following introduction to the questions:

Think about a recent situation at school when you felt you did not fully cope or failed to cope. This could be an examination, an academic presentation, achieving a grade, or something else that you consider important but were unable to manage. On a scale from 1 = “does not fit at all” to 5 = “fit very well,” evaluate how well the statements below correspond to what you usually do after experiencing a situation that you have not mastered.

#### Perceived Meaningfulness of School

To measure the perceived meaningfulness of school, a scale from Thuen and Bru (2009) was used. This scale contained three items on how useful, meaningful, and interesting students find schoolwork. The items were rated on a 10-point anchored scale (1 = *completely untrue*, 10 = *completely true*).

### Statistics

Initially, we scrutinized the distribution of all included items. Furthermore, we estimated the means, standard deviations, reliability, and intercorrelations of all latent variables. To investigate the construct validity of the MHLS-9, we performed confirmatory factor analysis (CFA) and followed the standards for educational and psychological testing in examining the scale’s relationships to conceptually related constructs (American Educational Research Association [AERA] et al., 2014, p. 27).

On the theoretical basis, we expected the MHLS-9 to be positively related to the variables “well-being–positive affect,” “sense of mastery,” “coping strategies–task-focused,” “coping strategies–help seeking,” and “perceived meaningfulness of school” and to be negatively related to “well-being–negative affect.”

To evaluate the overall fit of the CFA and correlation models, we used χ^2^ statistics and the following fit indices reported by the Mplus software (Muthén & Muthén, 2017): the comparative fit index (CFI), root mean square error of approximation (RMSEA), and standardized root mean square residual (SRMR). After reviewing the literature on cutoff criteria for goodness of fit (Hu & Bentler, 1998, 1999; Marsh et al., 2004), we adopted the following criteria for model evaluation: CFI ≥ .90, RMSEA ≤ .08, and SRMR ≤ .09, which indicated an acceptable fit, whereas CFI ≥ .95, RMSEA ≤ .06, and SRMR ≤ .06 indicated a good fit.

We also computed factor determinacy scores (FDSs) and internal consistency reliability (ω). FDS is a validity coefficient representing a correlation between the estimated factor score and the true factor score; it ranges from 0 to 1 and describes how well the factor in question is measured. For a scale to be considered reliable and have good factor determinacy, the FDS should be ≥.80 and preferably >.90, whereas ω should be ≥.70 (Brown, 2015).

## Results

Initially, we explored the nine items regarding distributional properties (Table 1). Five items had kurtosis close to 1 (−0.84 to −1.01). Therefore, we decided to use robust maximum likelihood estimation.

**Table 1:** Descriptive Statistics and Factor Loadings for the Mental Health Learning Scale (MHLS)

*Item*	*Wording*	*M*	*SD*	*Skew*	*Kurt*	*FL*
mhls1	Handling stressful situations in a good manner	2.78	1.18	0.00	−0.91	0.69
mhls2	Believing in yourself	3.01	1.17	−0.10	−0.86	0.81
mhls3	Having good sleep routines	2.71	1.25	0.16	−1.01	0.53
mhls4	Making decisions based on your own will	3.42	1.08	−0.42	−0.40	0.71
mhls5	Setting limits for your own actions	3.49	1.05	−0.49	−0.24	0.65
mhls6	Feeling that you belong in a community	3.45	1.10	−0.44	−0.44	0.73
mhls7	Mastering your own negative thoughts	2.81	1.21	0.08	−0.89	0.79
mhls8	Setting limits for what is OK for you	3.54	1.10	−0.52	−0.34	0.70
mhls9	Feeling valuable regardless of your own accomplishments	3.07	1.20	−0.15	−0.84	0.80

*Note.* Skew = skewness; Kurt = kurtosis; FL = standardized factor loading with a single factor model.

We then specified a one-factor model with the nine MHLS-9 items, which fitted the data moderately: χ^2^(27) = 454, *p* < .001; CFI = .935; RMSEA = .089, 90% confidence interval (CI) = [.082, .096]; SRMR = .037. While the SRMR was good and the CFI adequate, the RMSEA was less acceptable, probably because it evaluates less-complex models more strictly than the other fit indices. To search for the cause of the misfit, we examined the Mplus modification index; the results indicated that Items 5 and 8 had a certain amount of common variance outside the model. After scrutinizing the content and wording of these items, we found it reasonable to test a modified model in which these items’ residuals were correlated. The revised model showed an improved fit: χ^2^(26) = 291, *p* < .001; CFI = .959; RMSEA = .071, 90% CI = [.064, .079]; SRMR = .030. The factor loadings varied from 0.53 to 0.81 (Table 1). This model also revealed excellent factor determinacy (.95) and scale reliability (ω = .91).

Next, we examined the current scale’s correlation using other theoretically related concepts (e.g., well-being and sense of mastery) by specifying and testing a latent correlation model using Mplus. The model fitted the data well: χ^2^(412) = 2,345, *p* < .001; CFI = .930; RMSEA = .048, 90% CI = [.046, .050]; SRMR = .044. The MHLS-9 was positively correlated with well-being–positive affect and negatively with well-being–negative affect (Table 2, boldface). Furthermore, as expected, the MHLS-9 was positively correlated with sense of mastery, coping strategies–task-focused, coping strategies–help-seeking, and perceived meaningfulness of school, establishing the convergent validity of the measure.

**Table 2:** Intercorrelations, Means, Standard Deviations, and Reliability of the Latent Variables (*N* = 2,012)

*Variable*		1	2	3	4	5	6	7
1	Mental health learning in school	—						
2	Well-being–positive affect	**.60**	—					
3	Well-being–negative affect	−**.44**	−.64	—				
4	Sense of mastery	**.49**	.56	−.76	—			
5	Coping strategies–task-focused	**.58**	.46	−.23	.34	—		
6	Coping strategies–help-seeking	**.52**	.40	−.17	.28	.62	—	
7	Perceived meaningfulness of school	**.50**	.49	−.26	.35	.59	.44	—
	Variable range	1–5	1–10	1–10	1–10	1–5	1–5	1–10
	*M*	3.14	6.03	3.93	6.87	3.13	2.79	4.91
	*SD*	0.86	1.97	1.90	2.01	0.85	0.97	2.15
	Reliability (ω)	.91	.84	.83	.90	.80	.82	.89

*Note.* Correlations related to the MHLS-9 marked in boldface. MHLS-9 = Mental Health Learning Scale–9.

*p* < .001 for all correlations.

## Discussion

The purpose of the current study was to modify and test the modified version of the MHPK-10 to assess adolescents’ perceived *learning* of factors promoting good mental health at school, rather than self-reported and subjective knowledge levels. Findings suggest that we successfully adapted the MHPK-10 for use in pedagogical settings with the new nine-item MHLS (MHLS-9). Statistical testing was performed to evaluate the modifications, to determine if the applied modifications solved the previous ceiling effects, and to evaluate the factor structure of the modified scale. In contrast to the original MHPK-10 by Bjørnsen et al. (2017), the MHLS-9 shows no signs of ceiling effects, indicating that the modifications resolve the ceiling effect found among Norwegian adolescents when using the original instrument. The new MHLS-9 is feasible for measuring adolescent learning about factors promoting good mental health among ninth graders in Norway. In line with its predecessor, the MHPK-10, the new MHLS-9 functions as a unidimensional scale (Bjørnsen et al., 2017). Furthermore, significant factor loadings, good model fit, and significant correlations with the other included constructs in the expected directions support the construct validity of the new scale.

The MHLS-9 is a tool for schools and school health services to use in collaboration to promote good mental health. Many aspects of society, including health and education, are giving increased attention to adolescent mental health, and further actions are needed following the consequences to adolescent mental health following the COVID-19 pandemic (Fazel & Hoagwood, 2021). As we recover from the pandemic, studies have already demonstrated rising rates of internalizing disorders, such as depression and anxiety, in adolescents who were healthy prior to the pandemic (Cohen et al., 2021). The COVID-19 pandemic is expected to have a substantial impact on adolescent mental health; thus, promoting good mental health in this population is as important as ever. Schools and school health services have a central position in promoting good mental health and in addressing the consequences of the virus beyond physical health. Increasing MHL, specifically positive MHL by learning about factors promoting good mental health, for example, normal emotional variations, relatedness, autonomy, and mastering, is one way of promoting mental health in this population. However, to focus mental health education to the needs of adolescents and to evaluate the provided education, valid and reliable measures are needed. The MHLS-9 is one such measure, able to assess knowledge gaps among adolescents to focus the education toward the needs in, for example, a classroom setting and to evaluate the health education provided to the adolescent population.

Schools are an optimal setting for mental health promotion, as most adolescents can be reached through a school. However, both implementations and evaluations of mental health–promoting education are scarce (Eschenbeck et al., 2019). In Norway, the renewal of the Norwegian curriculum to include public health and life skills gave direction to schools and health care to work more closely together to promote students’ mental health. The MHLS-9 is one result of education and health working together, as we adapted the MHPK-10, developed for school health services, into a measure more relevant to the pedagogical personnel at schools. The MHLS-9 is potentially an instrument that both school health services and pedagogical personnel at schools may use to focus and evaluate mental health promotion initiatives in accordance with the school curriculum and the national guidelines for school health services (Norwegian Directorate of Health, 2017). The MHLS-9 can thus be an addition to the instruments used to evaluate the activities introduced by the curriculum renewal’s addition of public health and life skills in Norway.

## Limitations

Two strengths of the current study are the large sample size and the sampling process, where ninth graders across Norway were included. Moreover, the new MHLS-9 is derived from an MHL instrument originated in theory (Bjørnsen et al., 2017). The current study provides a sound psychometric evaluation of the MHLS-9; however, some caution should be used in interpreting the results. The current study only validates the MHLS-9 for use among ninth graders; thus, use in other age groups should be done using caution. Furthermore, the MHLS-9 measures self-reported and perceived knowledge learned at school, rather than measuring actual knowledge, and it may therefore introduce bias from the students over- or underestimating their own learning. However, the students’ perceived learning is important information in both planning and evaluating educational initiatives aiming at increased knowledge of how to obtain and maintain good mental health. Finally, a good model fit does not guarantee that the “true model” is obtained, and one should consider that alternative models might fit the data as well as the identified model (Bollen, 1989).

## Implications and further research

The MHLS-9 is an improved measure over the MHPK-10 for use in school settings. The MHLS-9 enables researchers and practitioners to evaluate and more precisely focus MHL interventions in schools by addressing students’ understanding of how to obtain and maintain good mental health using a short, reliable, and valid measure. The MHLS-9 is a valuable contribution to the field of MHL research, enabling the measurement of the perceived learning of factors promoting good mental health. The MHLS-9 is an addition to the toolbox of both professionals in schools and those in school health services. Furthermore, using the MHLS-9 may outline focus areas where students are scoring low for tailored mental health promotion education in schools. Further studies are recommended to evaluate the MHLS-9 in other samples and age groups. The work on defining and assessing MHL in the context of children and adolescents’ health is still in development, and as health and learning are related, we see great benefits in connecting health promotion services, for example, school health services and education, by introducing the MHLS-9, a valuable contribution to the MHL knowledge base.

## Conclusion

Overall, this study found support for introducing a revised version of the MHPK-10: a short, reliable, and valid measure of adolescents’ self-reported learning of factors promoting good mental health at school—the MHLS-9.

## Notes

1. A ceiling effect was not found in a Turkish study validating the 10-item Mental Health-Promoting Knowledge (MHPK-10; Mercan & Coşkun, 2022).
